# DNA Strand-Displacement Temporal Logic Circuits

**DOI:** 10.1021/jacs.2c04325

**Published:** 2022-07-02

**Authors:** Anna P. Lapteva, Namita Sarraf, Lulu Qian

**Affiliations:** † Bioengineering, California Institute of Technology, Pasadena, California 91125, United States; ‡ Computer Science, California Institute of Technology, Pasadena, California 91125, United States

## Abstract

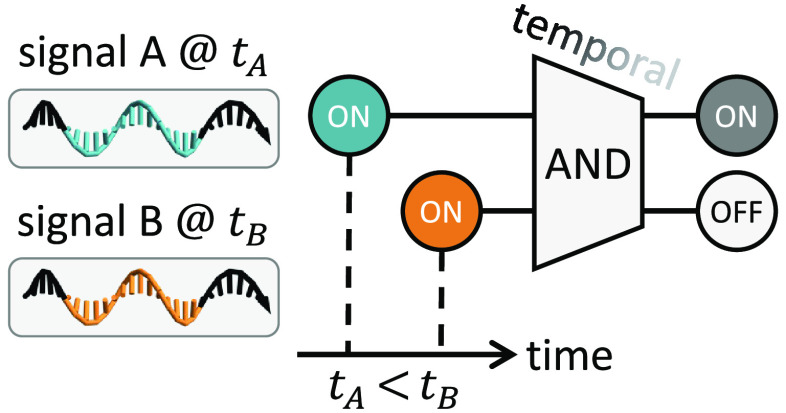

Molecular circuits capable of processing temporal information are essential for complex decision making in response to both the presence and history of a molecular environment. A particular type of temporal information that has been recognized to be important is the relative timing of signals. Here we demonstrate the strategy of temporal memory combined with logic computation in DNA strand-displacement circuits capable of making decisions based on specific combinations of inputs as well as their relative timing. The circuit encodes the timing information on inputs in a set of memory strands, which allows for the construction of logic gates that act on current and historical signals. We show that mismatches can be employed to reduce the complexity of circuit design and that shortening specific toeholds can be useful for improving the robustness of circuit behavior. We also show that a detailed model can provide critical insights for guiding certain aspects of experimental investigations that an abstract model cannot. We envision that the design principles explored in this study can be generalized to more complex temporal logic circuits and incorporated into other types of circuit architectures, including DNA-based neural networks, enabling the implementation of timing-dependent learning rules and opening up new opportunities for embedding intelligent behaviors into artificial molecular machines.

## Introduction

Temporal information processing involving the relative timing of signals is powerful and pervasive in biological systems, underlying a variety of phenomena across scales. At the organism level, animals use the relative timing of auditory stimuli to compute spatial information for hunting.^1^ At the cellular level, neurons use the relative timing of pre- and postsynaptic spikes to determine synaptic modification for learning.^2^ At the molecular level, genetic regulatory networks use the relative timing of transcription factors to control gene expression for responding to stress.^3^ It has been articulated that understanding the design principles of temporal information-processing circuits is critical both for answering fundamental questions regarding cellular dynamics and for engineering molecular systems with embedded controls.^4^

Synthetic molecular circuits capable of processing time-dependent information have been explored in theory^5−7^ and experiments.^8−11^ Two common strategies for detecting relative timing are cross inhibition^4,11^ and temporal memory.^5,10^ Cross inhibition entails the production of a circuit output when the first input signal arrives and simultaneous generation of inhibitors that target subsequent inputs; this strategy allows for *n* output decisions with *n* input signals. By contrast, temporal memory does not yield any circuit output until all input signals have arrived, while memories of earlier inputs lead to distinct circuit responses to later inputs; this strategy allows for *n*! output decisions with *n* input signals, resulting in combinatorial regulation based on the sequence of a small number of inputs.

Prior experimental demonstrations of temporal information processing utilized recombinase-based genetic circuits,^8,9^ polymerase-based primer exchange reactions,^10^ and DNA strand-displacement circuits.^11^ The mechanism of DNA strand displacement^12^ is capable of implementing complex computation^13,14^ and universal chemical kinetics.^15−17^ A variety of signals including small molecules, RNA, proteins, electricity, heat, and light can be converted to and from DNA signals, enabling excellent interfaces with biological and nonbiological systems for applications in chemistry, medicine, and materials.^18−21^ Building on the success of DNA strand-displacement circuits, here we demonstrate the strategy of temporal memory in DNA-only systems. Unlike the previous demonstration in DNA strand-displacement circuits using cross inhibition, we show that the implementation of temporal memory not only is compatible with combinatorial regulation but also enables temporal information to be incorporated into Boolean logic computation. We also show that the timing-based logic computation can be carried out using simple, two-stranded gate motifs, which we have argued to be important for the scalability of DNA strand-displacement circuits^22,23^ due to their robustness to synthesis errors and structural malformation.^24,25^

## Results and Discussion

**Figure 1:**
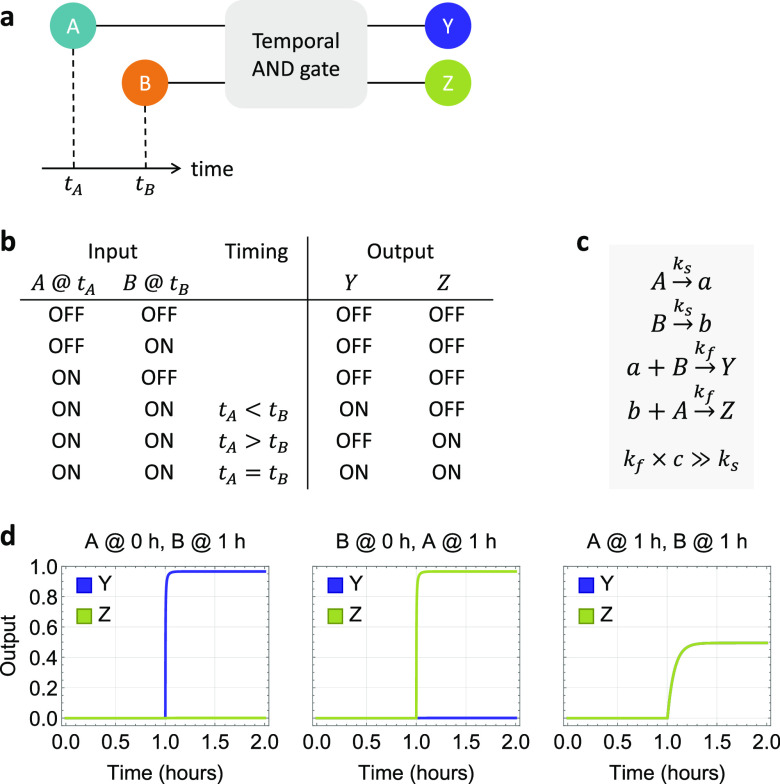
Concept and chemical reaction network implementation of temporal logic circuits. (a) Abstract circuit diagram, (b) truth table, (c) chemical reaction network implementation, and (d) simulations of a two-input temporal AND gate. *c* is the concentration of input signals *A* and *B*. Simulations of output signals *Y* and *Z* are shown as relative concentrations to *c* over time, where *c* = 100 nM, *k*_s_ = 0.002/s, and *k*_f_ = 2 × 10^6^ /M/s.

Here we define a temporal logic gate as follows (Figure 1a): each input (e.g., *A*) contains both a logic value (i.e., ON or OFF) and timing information (e.g., *t*_*A*_), while distinct outputs represent the recognition of specific combinations of signal values and their relative timing (e.g., *A* = ON, *B* = ON, and *t*_*A*_ < *t*_*B*_). Compared to a regular two-input AND gate, a two-input temporal AND gate has two more input combinations and one more output signal that distinguish three unique situations of relative timing when both *A* and *B* are ON (Figure 1b). The temporal gate is logically symmetric, that is, AND(*A@t*_*A*_, *B@t*_*B*_) = AND(*B@t*_*B*_, *A@t*_*A*_), and so are the implementations discussed below.

An abstract chemical reaction network implementation of the two-input temporal AND gate is shown in Figure 1c. The first pair of reactions converts an input signal *A* (or *B*) to a memory species *a* (or *b*). The second pair of reactions allows a historical and a current signal *a* and *B* (or *b* and *A*) to collectively produce an output signal *Y* (or *Z*). When the first input arrives, no memory species is available, and thus only one of the first two reactions could take place. If the second input arrives after the first input has been fully converted to a memory, it will yield the desired output by reacting with the memory. The second pair of reactions takes place at a much faster rate than the first pair so that the second input will preferentially trigger output production rather than becoming a memory itself. If neither or only one input is present, no output will be produced, ensuring desired logic function of an AND gate. If both inputs are present and arrive at the same time, they will both start by converting to memory species, but once enough memory has accumulated, they will each react with a memory to produce a distinct output (Figure 1d).

**Figure 2:**
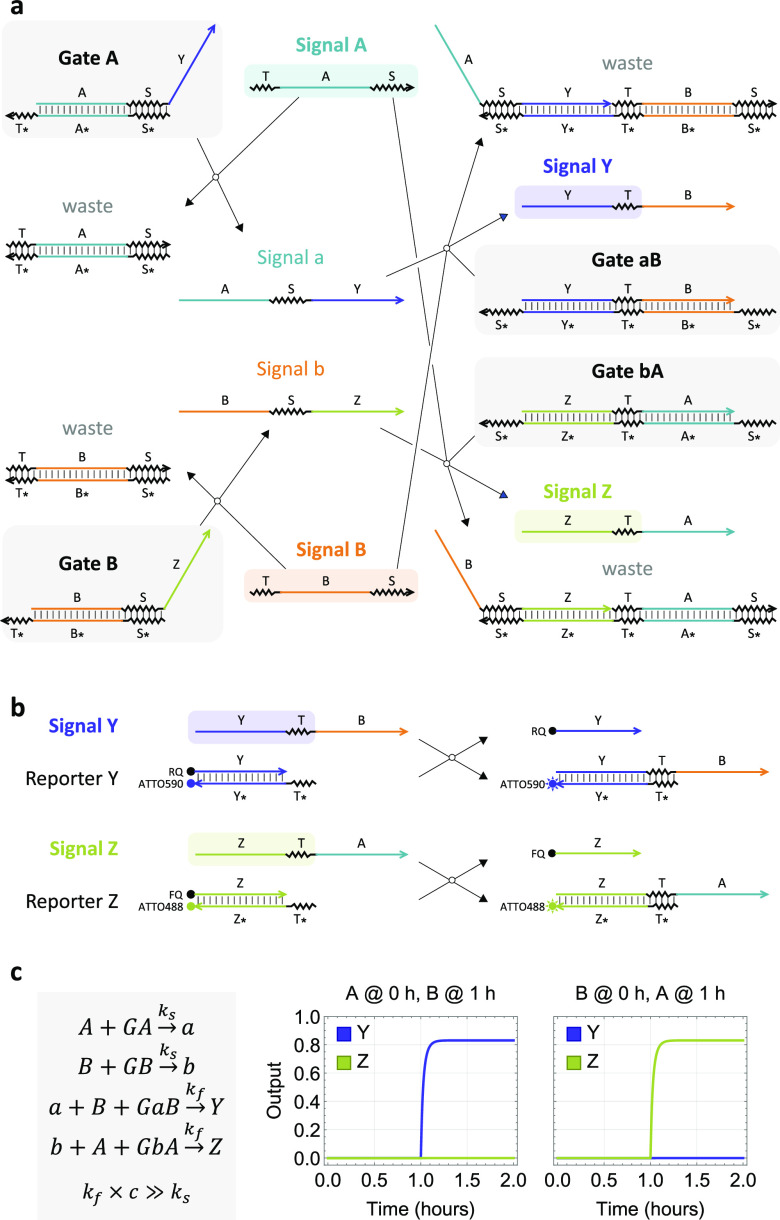
DNA strand-displacement implementation of a two-input temporal AND gate. (a) Reaction pathways. (b) Reporting mechanism. Zigzagged and straight lines indicate short toehold and long branch migration domains, respectively. Asterisks in domain names indicate sequence complementarity. Gray boxes highlight gate species composing the circuit. Colored boxes in signal strands highlight functional domains that participate in downstream reactions. (c) Simulations. *c* is the concentration of input signals *A* and *B*. Output signals *Y* and *Z* are shown as relative concentrations to *c* over time, where *c* = 100 nM, *k*_s_ = 10^5^/M/s (estimated strand displacement rate with a 5-nt toehold), and *k*_f_ = 2 × 10^13^/M^2^/s (estimated cooperative hybridization rate with a 7-nt toehold). Reporting reactions are not shown here but were included in simulations (Supplementary Note S2). Gates and reporters were in 20% and 50% excess compared to inputs, respectively.

Each of the abstract chemical reactions shown in Figure 1c can be implemented with a DNA strand-displacement reaction, where a gate species is designed to facilitate desired signal recognition and output production (Figure 2a). Each input signal is represented by a single-stranded DNA with two toeholds flanking a branch migration domain. Toehold T on the 5′ end of an input strand initiates a displacement reaction with an upstream gate, releasing a previously inhibited memory strand by uncovering its toehold S for downstream reactions. Upon release of the memory strand, the input strand becomes bound to the gate bottom strand, forming a waste molecule that has no open toeholds. When a second input strand arrives, together with the memory strand they react with a downstream gate by cooperative hybridization,^26^ initiating branch migration from two sides of the gate molecule via toehold S. When both junctions of branch migration meet at the middle of the gate, an output strand will be released. Despite having the same branch migration domain, the toehold T on the opposite side of the branch migration domain allows the output strand to participate in other reactions that the memory strand cannot.

For achieving output production faster than memory formation, it is desired to design a longer toehold S than toehold T. For example, S and T with 7 and 5 nucleotides, respectively, can give rise to a roughly 20-fold rate difference^27−29^ between the bimolecular steps of an input strand reacting with the upstream and downstream gates. In addition to allowing the input to react with the two gates at distinct rates, toehold S also ensures that memory formation is irreversible so that the relative timing information will be locked in place.

To demonstrate the composibility of the DNA strand-displacement temporal logic gate and to quantitatively understand the kinetics of the molecular behavior, two standard reporters with distinct fluorophores and quenchers can be used to simultaneously detect the production of the two output strands (Figure 2b). These reporters were previously used in complex DNA logic circuits^24^ and neural networks,^23^ suggesting that the output signals in the temporal logic circuit can readily serve as input signals to other strand-displacement circuits.

**Figure 3:**
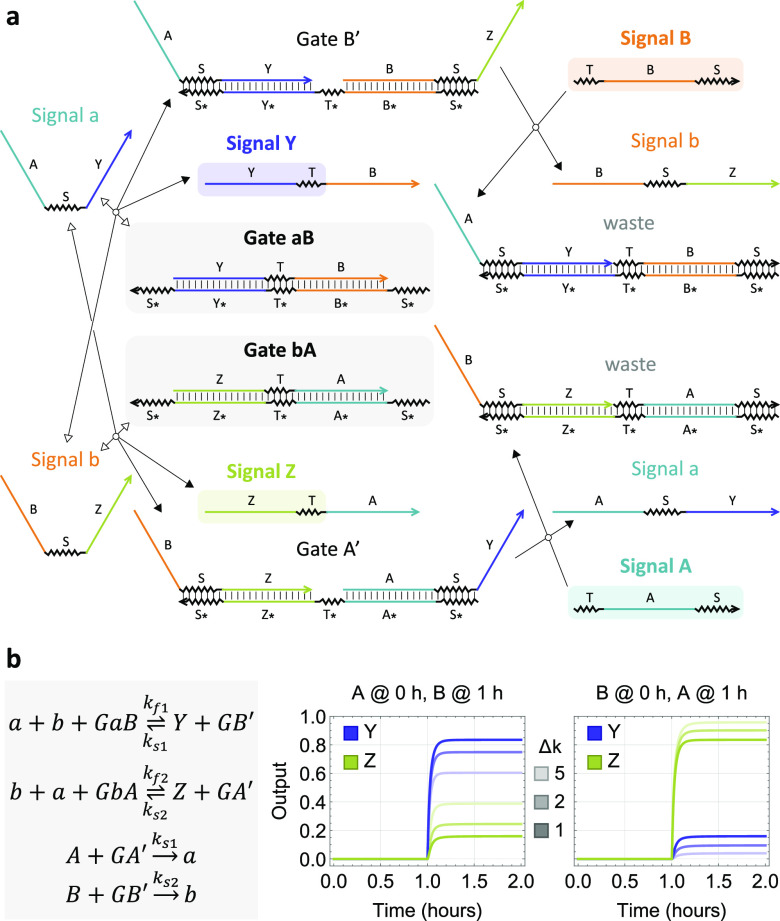
Crosstalk between two reaction pathways. (a) Crosstalk reactions. Forward and backward reactions are indicated as filled and open arrows, respectively. (b) Simulations of desired reactions shown in Figure 2c together with crosstalk reactions shown here, where input concentration *c* = 100 nM, *k*_s2_ = 10^5^/M/s, *k*_f2_ = 2 × 10^13^/M^2^/s, *k*_s1_ = *k*_*s*2_/Δ*k*, and *k*_f1_ = *k*_f2_/Δ*k*. The darkest to lightest trajectories correspond to simulations with no difference and a 2-fold and a 5-fold difference between the two pairs of rate constants, respectively.

In the ideal case, only one of the two reaction pathways involving an upstream and a downstream gate shown in Figure 2a will become active if one input signal arrives before the other. However, the rate difference favoring output production does not fully prevent the second input signal from reacting with its upstream gate to release the second memory strand. Moreover, a pair of competing reactions will occur when both memory strands are present, creating a crosstalk between these two reaction pathways; the two memory strands can reversibly react with both downstream gates as two cooperative inputs, resulting in undesired output (Figure 3a).

**Figure 4:**
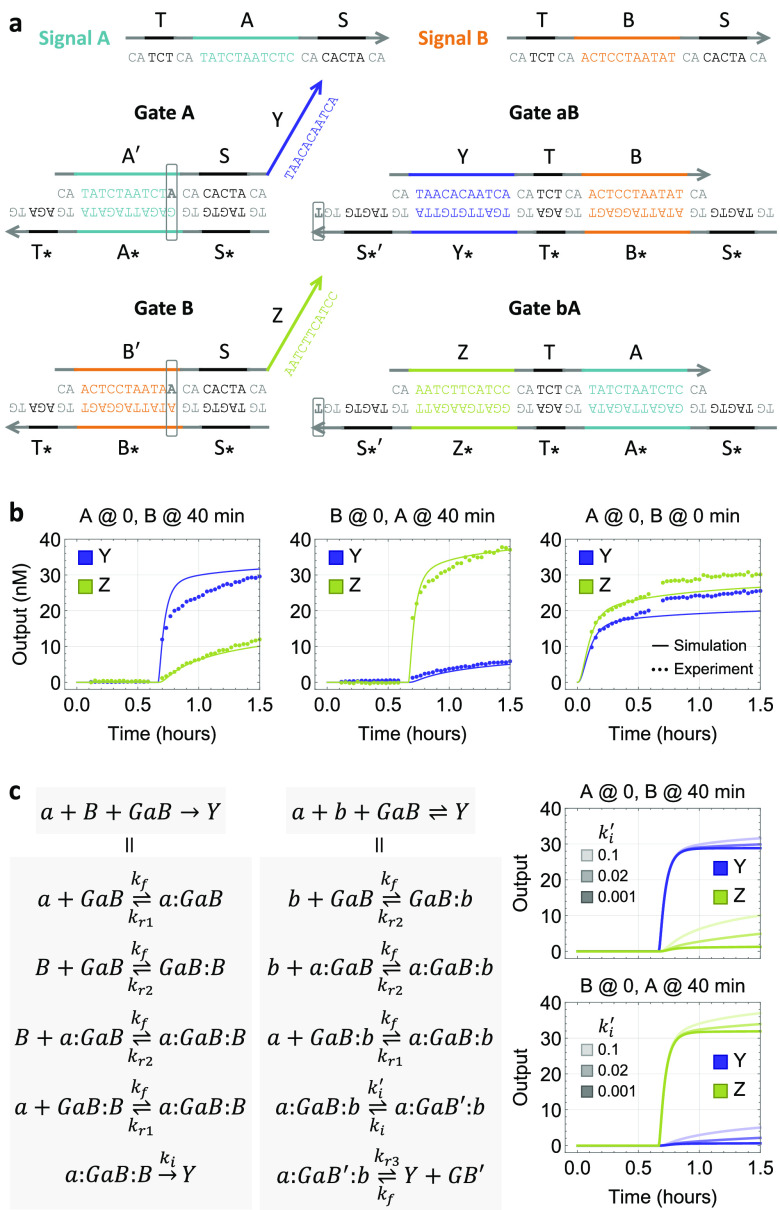
Characterization of circuit behavior. (a) Sequence design that reduces crosstalk by utilizing mismatches. A two-nucleotide clamp domain (colored in gray) is used for reducing undesired leak reactions between gates.^22^ Each toehold domain consists of a core sequence (colored in black) and a clamp. (b) Simulation and fluorescence kinetics data. All gates, reporters, and inputs were at 100, 150, and 90 nM, respectively. (c) Simulations with varying branch migration rates. A detailed model that includes binding (with rate *k*_f_), toehold dissociation (with rate *k*_r_), and branch migration (with rate *k*_i_) steps. Two trimolecular reactions are shown here as examples, but all four trimolecular reactions in Figures 2c and 3b were converted to the detailed model in simulations (a full list of reactions and rate constants are shown in Supplementary Note S2).

We investigated the impact of the crosstalk in simulations. Compared to the ideal system behavior without crosstalk (Figure 2c), the output that is supposed to turn ON largely remained the same, but the output that is supposed to stay OFF had a mildly elevated signal level at reaction completion (Figure 3b, darkest trajectories). The ON–OFF separation could become worse if reaction rates in the two pathways are not equal (Figure 3b, lighter trajectories), which we will show later in experiments. It is possible to use a pair of translator gates^13^ to remove the partial sequence of an input from the memory strand and eliminate the crosstalk. However, in the interest of keeping the implementation as simple as possible, we explored an alternative solution using a mismatch in each memory strand (Figure 4a). The mismatch is located near the 3′ end of the branch migration domain in each upstream gate that releases a memory strand. The double-stranded domains on both sides of the mismatch are long enough to ensure structural stability of the gate (Figure S1). As shown in a previous study,^30^ elimination of a mismatch that is sufficiently distant from the toehold (e.g., position 13 in the branch migration domain) results in roughly the same strand displacement rate as no mismatches, suggesting that the mismatch should not affect the rate with which the memory strand is released. However, creation of a mismatch near the toehold (e.g., position 3 in the branch migration domain) can result in over 100 times slower kinetics when the toehold is sufficiently short (e.g., 7 nt),^31^ suggesting that the mismatch should reduce the rate of crosstalk by slowing down branch migration that leads to undesired output production.

An additional benefit of the mismatch is that it allows for an extended toehold on the cooperative gates (Figure 4a). Without mismatches, if the toehold is too long, both input strands can spuriously bind to the wrong side of the gates and temporarily inhibit themselves and the gates; this effect is known as toehold occlusion, which could slow down the circuit and introduce undesired system behaviors.^17,22^ With the mismatch, the occlusion will not become any worse when the toehold is extended by one nucleotide, allowing the desired reaction between a memory strand and a gate to be faster.

Fluorescence kinetics experiments confirmed the impact of the crosstalk and revealed a rate difference between the two reaction pathways favoring the production of output *Z* (Figure S3a). With a mismatch in each memory strand, the production of undesired output was significantly slowed down (Figure S3b). Furthermore, altering the sequence of a branch migration domain reduced the difference between the two pathways (Figure S3c). Specifically, domain B was changed to have a more even spread of cytosines similar to the other three branch migration domains *A*, *Y*, and *Z*. Previous work has shown that even with the same toehold sequence, the effective rate of strand displacement can differ 3-fold with varying branch migration sequences.^23^ This difference could be larger in cooperative hybridization. A biophysical study would enable a better understanding of how the rate depends on both branch migration sequences. Until then, each reaction rate of interest could be measured in isolation for a library of branch migration sequences, and a subset of them with the most similar rates could be chosen for building a circuit with balanced output production.

Overall, the temporal logic circuit correctly computed a two-input AND function (Figure 4b): when only one of the inputs was present, both outputs remained OFF; when both inputs were present, three unique output combinations (*Y* = ON and *Z* = OFF, *Y* = OFF and *Z* = ON, *Y* = ON and *Z* = ON) were observed depending on the three distinct relative timings of inputs (*t*_*A*_ < *t*_*B*_, *t*_*A*_ > *t*_*B*_, *t*_*A*_ = *t*_*B*_).

Noticeably, the simulation shown in Figure 3b did not predict the experimental observation in Figure 4b, where the output concentrations continued to increase during the course of the experiment without reaching a steady state. To gain a better understanding of the molecular behavior, we investigated a more detailed model, similar to the three-step model previously developed to understand noncooperative strand displacement.^29^ Here, each irreversible trimolecular reaction in the desired pathways (Figure 2c) is replaced by four bimolecular reactions that model binding and toehold dissociation and one unimolecular reaction that models branch migration (Figure 4c, left). When one strand is bound to either side of a cooperative gate by a toehold (*a*:*GaB* or *GaB*:*B*), it can reversibly dissociate. A second strand can reversibly bind to the same cooperative gate on the opposite side, leaving both toeholds occupied (*a*:*GaB*:*B*). When both strands branch migrate to the middle point of the cooperative gate, an output strand (*Y*) will be irreversibly released. Similarly, each reversible trimolecular reaction in the crosstalk pathways (Figure 3b) is replaced by six bimolecular and unimolecular reactions, the first of which already exists in the desired pathways (Figure 4c, middle). Instead of a memory strand binding to the left side and an input strand binding to the right side of a cooperative gate, here both sides of the gate will be occupied by memory strands (*a*:*GaB*:*b*). Branch migration will create a mismatch in the forward direction (with rate *k′*_i_) and eliminate a mismatch in the backward direction (with rate *k*_i_). When both strands branch migrate to the middle point (*a*:*GaB*′:*b*), toehold dissociation is needed to release the output strand, which can reversibly bind to the three-stranded complex with an open toehold in the middle (*GB*′). This model omits branch migration steps that occur on one but not both sides of a cooperative gate, which would require an additional seven reactions as used for modeling a cooperative catalyst.^32^ Nonetheless, by modeling binding and toehold dissociation on either side of a cooperative gate, it captures two important facts that the abstract model with trimolecular reactions does not: one signal will be temporarily consumed even if the other signal is not present; when the gate is in excess, two strands can bind to the opposite sides of two copies of the gate and not produce any output (a more detailed explanation is given in Figure S2). Importantly, the lumped branch migration step still allows for the overall impact of mismatches to be modeled.

With the detailed model, simulation semiquantitatively reproduced the experimental data (Figure 4b). The OFF trajectories agreed well, while the ON trajectories still had some differences. It is yet to be explored whether an even more detailed model at the base-pair level^33^ would allow for a better explanation. Simulations also suggested that the branch migration rate in the crosstalk pathways would need to be further reduced from 0.1/s to 0.001/s in order to fully suppress undesired output production (Figure 4c, right). This could be achieved by altering the mismatch sequences (e.g., to C–C mismatches^31^), using more mismatches in a branch migration domain, or shortening the toehold.^33^

**Figure 5:**
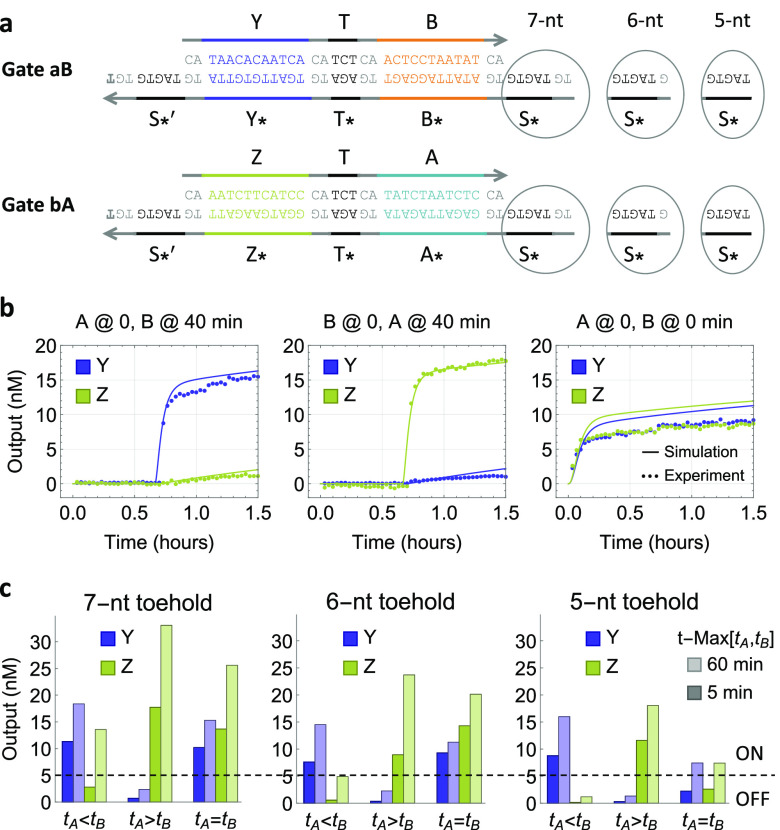
Demonstration of the two-input temporal AND gate with varying toehold lengths. (a) Sequence design with varying lengths of the S* toehold on the two cooperative hybridization gates. (b) Simulation and fluorescence kinetics data of the circuit with a 5-nt S* toehold. All gates, reporters, and inputs were at 100, 150, and 90 nM, respectively. (c) Output concentrations in experiments with varying toehold lengths. Darker and lighter bars correspond to output concentrations immediately (within 5 min, when the first data point was collected) and 1 h after the second input has arrived, respectively. Dashed line marks the separation between ON and OFF states.

We explored the option of shortening the toehold S* on the 5′ end of the bottom strand of the cooperative gates (Figure 5a). Because the crosstalk involves the memory strand binding to the wrong side of the gate, a shorter toehold on that side will create a stronger bias toward desired reactions. A trade-off is that the ON state of the output will also decrease with a shorter toehold due to an increased fraction of the input strand being converted to memory when it arrives second. We surveyed three distinct lengths of the toehold with fluorescence kinetics experiments. The 5-nt toehold exhibited the best separation between ON and OFF states in outputs (Figure 5b). Interestingly, the impact of rate difference between two reaction pathways was also significantly reduced with a shorter S* toehold (Figure S4). If we look only at the initial outputs right after the second input has arrived, the circuit computation could be considered correct for all three toehold lengths (Figure 5c). However, output *Z* failed to stay OFF over time when *t*_*A*_ < *t*_*B*_ for longer toeholds, indicating that the undesired crosstalk was not sufficiently inhibited. Decreased maximum signal in outputs was also observed in experiments with shorter toeholds, agreeing with the simulations (Figure 5b). If desired, an amplification step could be introduced to restore the output to a designated concentration, which has been demonstrated in DNA-based logic circuits.^13,22^ In general, these results suggest that a detailed model of cooperative hybridization is indeed important in guiding designs and experimental investigations for achieving robust circuit behavior.

**Figure 6:**
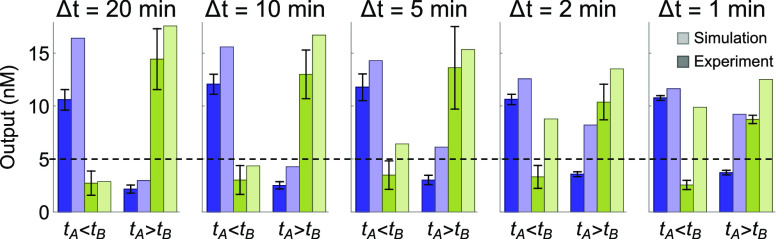
Varying time intervals between two inputs. Darker and lighter bars correspond to experiment and simulation of output concentration at 60 min after the second input was added, respectively. Δ*t* = |*t*_*A*_ – *t*_*B*_|. All gates, reporters, and inputs were at 100, 150, and 90 nM, respectively. Data were averaged over three independent experiments using varying qualities of the gate molecules (unpurified vs gel purified). Error bars indicate standard deviation of the mean. Dashed line marks the separation between ON and OFF states. An example set of kinetics data is shown in Figure S5.

Finally, we investigated the time resolution of the circuit. Intuitively, the second input should arrive after the first input has been converted to a memory strand, which will take 1/(ks*c) ≈ 100 s. The detailed model predicted that a minimum of Δ*t* = 5 min was needed for a clear ON–OFF separation where the output that turns ON is at least twice the concentration of the output that stays OFF, taking rate biases in output production into consideration (Figure 6). To evaluate the robustness of the circuit, fluorescence kinetics experiments were performed with unpurified and gel-purified gate molecules. Data averaged over three sets of experiments showed more robust system behavior than the simulation prediction: clear ON–OFF separation was observed even with Δ*t* = 1 min (Figure 6).

## Conclusions

To summarize, we have shown that a molecular information-processing circuit can be designed to store temporal information about molecular signals in a set of memory species that each encodes a unique relative timing of the signals. The circuit recognizes distinct combinations of signals, as well as their relative timing, and uses this information to make decisions for driving unique downstream processes. In principle, the two-input temporal AND gate can be generalized to more complex circuits. For example, a three-input temporal AND gate can be created by utilizing an increased number of memory species that each encodes the timing information on one or two historical signals (Figure S6). However, experimental demonstration of more complex circuits will be difficult, due to the fact that relatively small rate biases could significantly affect the system behavior. It will be desirable to explore more robust and efficient implementations, for example using polymers;^34^ instead of encoding the timing of signals in the identity of individual molecular species, the same information could be encoded in the order of monomers on a polymer. Essentially, converting temporal information to spatial information would allow the computation to be carried out by significantly fewer types of molecules. Other than producing output signals to control downstream reactions, the temporal information on molecular events could also be recorded in DNA sequences and readout using high-throughput sequencing techniques.^7^ Similarly, a DNA polymerase whose error rate depends on cation concentrations has been proposed as a molecular device for scalable recording of neural activities.^35−37^

With further developments, the strategy and implementation of temporal memory could be broadly employed in molecular circuits beyond logic computation. For example, bacteria utilize a transient memory to compare current and historical signal concentrations for recognizing attractant and repellent gradients in chemotaxis.^38,39^ More complex pattern recognition tasks can be performed by mammalian neural networks.^40^ Inspired by the fundamental importance of pattern recognition in biological systems, DNA strand-displacement circuits have been developed to carry out neural network computation.^23,41−43^ Incorporating temporal memories into these DNA circuits will enable the implementation of timing-dependent learning rules^2^ and open up new opportunities for embedding intelligent behaviors into artificial molecular machines.

## Supporting Information Available

Supporting methods, notes, figures, and DNA sequences (PDF)
