# Supplementary material for: DNA Strand-Displacement Temporal Logic Circuits
Source: J Am Chem Soc. 2022 Jul 2;144(27):12443–9. doi: 10.1021/jacs.2c04325 (PMC9284558; doi:10.1021/jacs.2c04325)
Supplement: Supplementary file 1 — ja2c04325_si_001.pdf [file ja2c04325_si_001.pdf]

DNA strand-displacement temporal logic circuits

Anna P. Lapteva[1†], Namita Sarraf[1†] and Lulu Qian[1,2⋆]
[1]*Bioengineering, and* [2]*Computer Science*
California Institute of Technology, Pasadena, CA 91125, USA
[†]*Equal contribution,* [⋆]*e-mail: luluqian@caltech.edu*

Supporting information

S1 Methods

S1.1 Sequence design

The temporal logic circuit involves DNA strands that consist of longer branch migration domains and shorter toehold domains, all of which are functionally independent. Therefore, sequence design was performed at the domain level.

Sequences must satisfy several criteria in order to qualify as candidates for the branch migration domain pool; these criteria were previously developed and intended to reduce undesired displacement reactions of mismatched invading strands.[1,2] To summarize, sequences must use a three-letter code (A, C, and T) to reduce secondary structures and undesired interactions, and there should be no more than 4 A's or T's in a row and no more than 3 C's in a row to reduce synthesis errors. Furthermore, the sequences must contain 30% to 70% C-content to ensure comparable melting temperatures. Finally, for any two sequences in the pool, at least 30% of the bases must be different, and the longest run of base pair matches must be at most 35% of the domain length. To prevent gate-gate leak caused by blunt end stacking, two-nucleotide "clamps" were also included in the sequence design. A distinct domain pool of sequences which satisfy the above conditions was generated via custom software.

To construct the temporal logic circuits demonstrated in this work, a pool of 15-nt branch migration domains was used, with every sequence containing a 2-nt clamp (CA) on both the 5' and 3' ends (which is the same domain sequence pool used for the Seesaw Compiler[3]). Toehold sequences also used the three-letter code. In order to selectively promote the formation of output over memory, we used a 5-nt toehold T for the reaction producing the memory species, and a 7-nt toehold S for the downstream reaction producing the output strands. Because toehold domains are unable to initiate strand displacement without compatible branch migration domains, we only require one of each kind of toehold in the circuits. Following the same clamp design in seesaw DNA circuits (supplementary note S8 in Reference 1), each toehold consists of a core sequence and a clamp. The 3-nt core of toehold T containing one C was taken from the previous work, while the 5-nt core of toehold S containing 2 C's was newly designed. All DNA strands in the circuit were generated through unique concatenations of branch migration domains and toehold domains (Table S1). Through NUPACK,[4] these designed strands were confirmed to not yield undesired secondary structures.

To reduce the crosstalk (i.e. a pair of competing reactions) shown in Figure 3a, a 1-nt mismatch was introduced in both Gate A and Gate B (Figure 4a). This mismatch permitted a favorable increase in the length of toehold S$*$ on the 3' end of the bottom strand of Gate aB and Gate bA; that toehold was increased from 7 to 8 nucleotides. We chose the mismatch sequence to be an A so as to prevent a G-T wobble pairing in Gate A and to allow the same toehold extension sequence T on both cooperative gates aB and bA. NUPACK analysis was performed to verify that the mismatch should not affect the structural stability of Gate A and Gate B at 25 °C and 100 nM concentration (Figure S1). The full set of sequences for the temporal logic circuit with mismatches is listed in Table S2. Note that sequences of Signal A, Signal B, Gate A-b, Gate B-b, Gate aB-t, and Gate bA-t are not affected by the above changes and thus are the same as in Table S1 (-t and -b indicate top and bottom strands in a gate, respectively).

In light of the preference for output Z observed in initial experiments, a second 15-nt sequence for domain B was chosen from the same domain pool to explore the impact of branch migration sequences on the overall rate of the reaction pathway. That sequence was chosen to have a more even spread of C's similar to the other three branch migration domains A, Y, and Z. The full set of sequences for the temporal logic circuit with the alternative B domain is listed in Table S3. Note that sequences of Signal A, Gate A-t, Gate A-b, Gate bA-t, and Gate bA-b do not involve domain B and thus are the same as in Table S2.

To further reduce the crosstalk between two reaction pathways, we investigated the performance of the system after shortening the length of toehold S$*$ on the 5' end of the bottom strand of Gate aB and Gate bA to 6 and

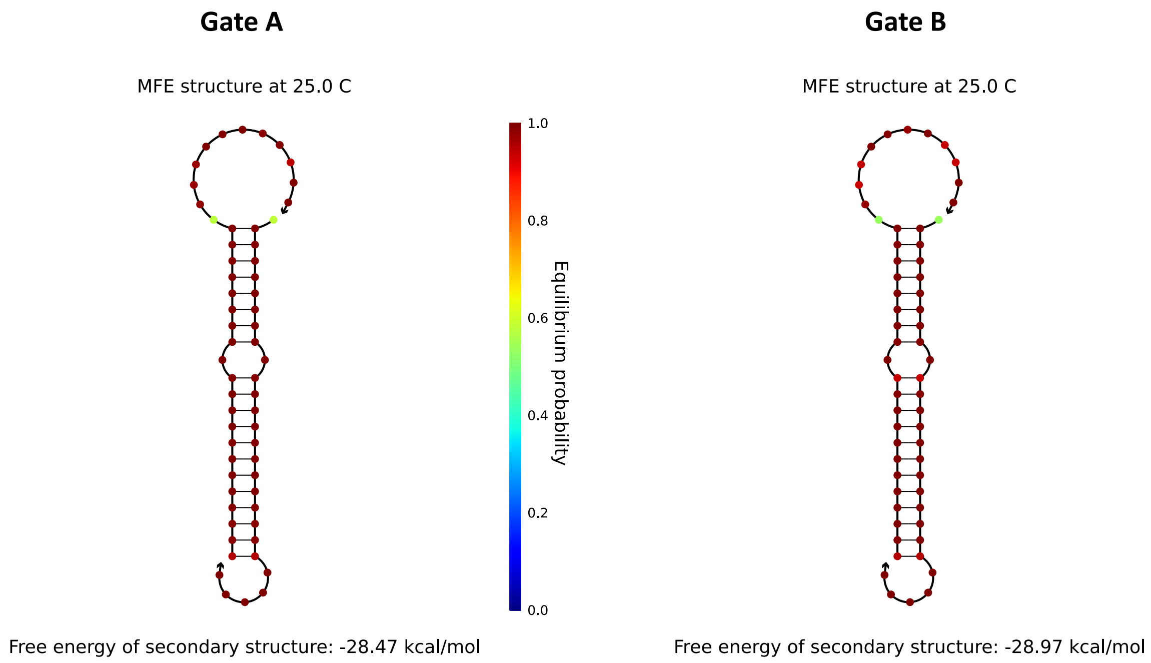

Figure S1: NUPACK analysis on the minimum free energy (MFE) structures of gates with mismatches.

5 nucleotides (Figure 5b). To evaluate the impact of toehold occlusion,[1,5] we also altered the sequence of toehold S in Signal A to be distinct from that in Signal B – this change turned out to not have any significance on the overall system behavior. The full set of sequences for the temporal logic circuit with varying toehold lengths is listed in Table S4. Note that sequences of Signal B, Gate B-t, Gate B-b, and Gate aB-t are not affected by the above changes and thus are the same as in Table S2. In all these sequence modifications (Tables S2, S3, and S4), the toehold and branch migration domains still conformed to the appropriate constraints established above.

Reporter sequences were taken from two previously designed reporters[2,6] and are listed in Table S5.

S1.2 Sample preparation

DNA oligonucleotides were synthesized by Integrated DNA Technologies (IDT). Strands without modifications were ordered with standard desalting, LabReady at 100 μM in IDTE buffer at pH 8.0. Strands with a fluorophore or quencher were ordered with high-performance liquid chromatography (HPLC) purification.

Annealing of complexes was performed in an Eppendorf thermocycler. The samples were cooled from 90 °C to 20 °C over the course of 90 minutes. At 90 °C, all strands should be single stranded with no secondary structures. As they cool down, they should preferentially form the designed complexes. The final buffer condition of all complexes was 1×TE with 12.5 mM Mg^{2+}.

Reporter complexes were not purified after annealing, but gate complexes were purified using polyacrylamide gel electrophoresis (PAGE). A 12% PAGE gel was run for 6 hours at 150 V in 1xTAE with 12.5 mM Mg^{2+}, with the exposed wire in the upper chamber of the gel box being cleared of salt every 1.5 hours. The relevant bands were incubated in 1×TE buffer with 12.5 mM Mg^{2+} for at least 24 hours.

The NanoDrop (Thermo Fisher) was used to quantify the concentrations of the complexes after purification. DNA absorbs UV light at 260 nm, so the measurement at that wavelength was used to calculate the concentration, normalized by the specific extinction coefficient of that species.

S1.3 Fluorescence kinetics experiments

Experiments were run with species concentrations specified in figure captions. Master mixes containing all shared circuit components were prepared in tubes and input mixes were added to a clear- and flat-bottomed 96-well plate. Master mixes were then added to the plate and mixed thoroughly. In instances where input signals were added

at different times, the second signal was added after one hour incubation at room temperature. The plate was then centrifuged and inserted into a microplate reader (Synergy H1, Biotek). Fluorescent readings were taken from the bottom of the plate at room temperature (approximately 22 °C). The excitation/emission wavelengths were 496/525 nm for ATTO488 and 598/629 nm for ATTO590.

S1.4 Data normalization

All raw fluorescence signal data were normalized to relative concentrations of output signals. Within each set of experiments, there was a negative control wherein the output signal stayed OFF, meaning that it remained at its minimum, as well as a positive control in which the output signal turned ON, meaning that it reached its maximum. The negative control included none of the input signals, while the positive control used an excess (2×) of input and memory strands to maximally trigger the release of output strand. For data normalization, the minimum output level was determined using the average of the first 5 data points of the negative control trajectory, while the maximum output level was determined from the average of the last 5 data points of the positive control trajectory. Ultimately, this process allows for meaningful quantitative analysis of the data, despite the use of different fluorophores.

S2 Modeling and simulation

All simulations were performed with mass-action kinetics using CRNSimulator.[7] In all reactions, Gate B, Gate aB, Gate bA, Gate A′, Gate B′, Reporter Y, and Reporter Z are represented as GA, GB, GaB, GbA, GA', GB', $RepY$, and $RepZ$, respectively.

In the detailed model, the following reactions were used to model the desired pathways:

$$
\begin{aligned}
A + GA &\xrightarrow{k_{s1}} a & B + GB &\xrightarrow{k_{s1}} b \\
a + GaB &\underset{k_{r1}}{\overset{k_f}{\rightleftharpoons}} a{:}GaB & b + GbA &\underset{k_{r1}}{\overset{k_f}{\rightleftharpoons}} b{:}GbA \\
B + GaB &\underset{k_{r2}}{\overset{k_f}{\rightleftharpoons}} GaB{:}B & A + GbA &\underset{k_{r2}}{\overset{k_f}{\rightleftharpoons}} GbA{:}A \\
B + a{:}GaB &\underset{k_{r2}}{\overset{k_f}{\rightleftharpoons}} a{:}GaB{:}B & A + b{:}GbA &\underset{k_{r2}}{\overset{k_f}{\rightleftharpoons}} b{:}GbA{:}A \\
a + GaB{:}B &\underset{k_{r1}}{\overset{k_f}{\rightleftharpoons}} a{:}GaB{:}B & b + GbA{:}A &\underset{k_{r1}}{\overset{k_f}{\rightleftharpoons}} b{:}GbA{:}A \\
a{:}GaB{:}B &\xrightarrow{k_i} Y & b{:}GbA{:}A &\xrightarrow{k_i} Z
\end{aligned}
\tag{S1}
$$

The following reactions were used to model the crosstalk:

$$
\begin{aligned}
a + GbA &\underset{k_{r2}}{\overset{k_f}{\rightleftharpoons}} GbA{:}a & b + GaB &\underset{k_{r2}}{\overset{k_f}{\rightleftharpoons}} GaB{:}b \\
a + b{:}GbA &\underset{k_{r2}}{\overset{k_f}{\rightleftharpoons}} b{:}GbA{:}a & b + a{:}GaB &\underset{k_{r2}}{\overset{k_f}{\rightleftharpoons}} a{:}GaB{:}b \\
b + GbA{:}a &\underset{k_{r1}}{\overset{k_f}{\rightleftharpoons}} b{:}GbA{:}a & a + GaB{:}b &\underset{k_{r1}}{\overset{k_f}{\rightleftharpoons}} a{:}GaB{:}b \\
b{:}GbA{:}a &\underset{k_i}{\overset{k_i'}{\rightleftharpoons}} b{:}GbA'{:}a & a{:}GaB{:}b &\underset{k_i}{\overset{k_i'}{\rightleftharpoons}} a{:}GaB'{:}b \\
b{:}GbA'{:}a &\underset{k_f}{\overset{k_{r3}}{\rightleftharpoons}} Z + GA' & a{:}GaB'{:}b &\underset{k_f}{\overset{k_{r3}}{\rightleftharpoons}} Y + GB' \\
A + GA' &\xrightarrow{k_{s2}} a & B + GB' &\xrightarrow{k_{s2}} b
\end{aligned}
\tag{S2}
$$

The following reactions were used to model the reporting mechanism:

$$
Y + RepY \xrightarrow{k_{s1}} FluorY \qquad Z + RepZ \xrightarrow{k_{s1}} FluorZ \tag{S3}
$$

The following reactions were used to model toehold occlusion:

$$
\begin{aligned}
A + GaB &\underset{k'_{r1}}{\overset{k_f}{\rightleftharpoons}} A{:}GaB & A + GbA &\underset{k'_{r1}}{\overset{k_f}{\rightleftharpoons}} A{:}GbA \\
A + GaB{:}B &\underset{k'_{r1}}{\overset{k_f}{\rightleftharpoons}} A{:}GaB{:}B & A + GbA{:}A &\underset{k'_{r1}}{\overset{k_f}{\rightleftharpoons}} A{:}GbA{:}A \\
B + GaB &\underset{k'_{r1}}{\overset{k_f}{\rightleftharpoons}} B{:}GaB & B + GbA &\underset{k'_{r1}}{\overset{k_f}{\rightleftharpoons}} B{:}GbA \\
B + GaB{:}B &\underset{k'_{r1}}{\overset{k_f}{\rightleftharpoons}} B{:}GaB{:}B & B + GbA{:}A &\underset{k'_{r1}}{\overset{k_f}{\rightleftharpoons}} B{:}GbA{:}A \\
b + GaB &\underset{k_{r1}}{\overset{k_f}{\rightleftharpoons}} b{:}GaB & a + GbA &\underset{k_{r1}}{\overset{k_f}{\rightleftharpoons}} a{:}GbA \\
b + GaB{:}B &\underset{k_{r1}}{\overset{k_f}{\rightleftharpoons}} b{:}GaB{:}B & a + GbA{:}A &\underset{k_{r1}}{\overset{k_f}{\rightleftharpoons}} a{:}GbA{:}A \\
A + GaB &\underset{k_{r2}}{\overset{k_f}{\rightleftharpoons}} GaB{:}A & B + GbA &\underset{k_{r2}}{\overset{k_f}{\rightleftharpoons}} GbA{:}B \\
A + a{:}GaB &\underset{k_{r2}}{\overset{k_f}{\rightleftharpoons}} a{:}GaB{:}A & B + b{:}GbA &\underset{k_{r2}}{\overset{k_f}{\rightleftharpoons}} b{:}GbA{:}B \\
a + GaB &\underset{k_{r2}}{\overset{k_f}{\rightleftharpoons}} GaB{:}a & b + GbA &\underset{k_{r2}}{\overset{k_f}{\rightleftharpoons}} GbA{:}b \\
a + a{:}GaB &\underset{k_{r2}}{\overset{k_f}{\rightleftharpoons}} a{:}GaB{:}a & b + b{:}GbA &\underset{k_{r2}}{\overset{k_f}{\rightleftharpoons}} b{:}GbA{:}b \\
A + GaB{:}b &\underset{k'_{r1}}{\overset{k_f}{\rightleftharpoons}} A{:}GaB{:}b & A + GbA{:}a &\underset{k'_{r1}}{\overset{k_f}{\rightleftharpoons}} A{:}GbA{:}a \\
B + GaB{:}b &\underset{k'_{r1}}{\overset{k_f}{\rightleftharpoons}} B{:}GaB{:}b & B + GbA{:}a &\underset{k'_{r1}}{\overset{k_f}{\rightleftharpoons}} B{:}GbA{:}a \\
b + GaB{:}b &\underset{k_{r1}}{\overset{k_f}{\rightleftharpoons}} b{:}GaB{:}b & a + GbA{:}a &\underset{k_{r1}}{\overset{k_f}{\rightleftharpoons}} a{:}GbA{:}a
\end{aligned}
\tag{S4}
$$

The following reaction rates were used in all simulations using the detailed model:

$k_f = 2 \times 10^6$ /M/s (rate of hybridization)

$k_{s1} = 2 \times 10^5$ /M/s (rate of strand displacement with a 5-nt toehold)

$k_{s2} = 10^6$ /M/s (rate of strand displacement with a 5-nt toehold incorporating an additional stacking bond in the middle of a cooperative gate)

$k_{r1} = 0.1$ or 0.02 /s (rate of dissociation for a 7-nt or 8-nt toehold on the 3' end of a cooperative gate)

$k'_{r1} = 0.1$ /s (rate of dissociation of signal A or B from the 3' end of a cooperative gate)

$k_{r2} = 0.1$, 1.5, or 5 /s (rate of dissociation for a 7-nt, 6-nt, or 5-nt toehold on the 5' end of a cooperative gate)

$k_{r3} = 0.5$ /s (rate of dissociation for a 5-nt toehold incorporating an additional stacking bond in the middle of a cooperative gate)

$k_i = 1$ /s (rate of branch migration in the desired pathways)

$k'_i = 1$ or 0.1 /s (rate of branch migration in the crosstalk pathways, without or with a mismatch)

In Figure S3a, to reproduce the bias in output production observed in the experimental data, k_{r2} (7-nt) for GaB was changed to 0.5 /s, which was 5 times faster than that for GbA. In Figures S3b and S4a, to better explain the experimental data, k_{r2} (7-nt) for GaB was changed to 2 /s and that for GbA was changed to 0.2 /s, while k_i for GaB was changed to 0.8 /s. In Figures 4b and S3c, k_{r2} (7-nt) for GaB was changed to 0.25 /s, which was 2.5 times

faster than that for GbA. In Figure S4b, k_{r2} (6-nt) for GaB was changed to 4 /s, which was 2.7 times faster than that for GbA, while k_i for GaB was changed to 0.8 /s. In Figures 5c and S4c, k_i for GaB was changed to 0.8 /s.

In all simulations, input concentrations were adjusted from 90 to 55 nM to better explain the experimental data. This is likely because the data was normalized based on triggered gate concentration, which was presumably higher than the input concentration due to the fact that gates were gel purified while inputs were unpurified. Another explanation is signal loss in non-catalytic reactions.[2]

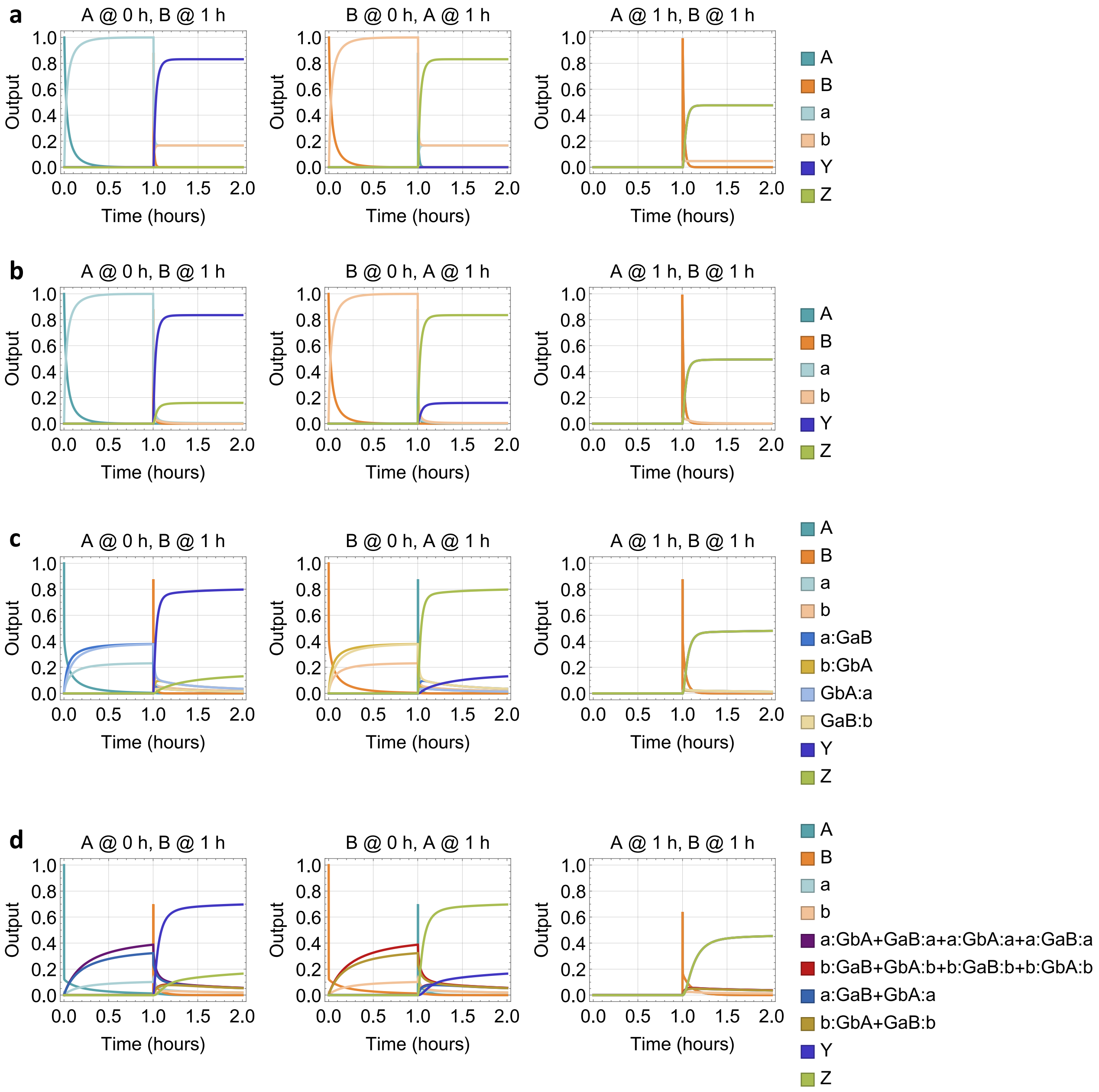

Figure S2: Simulation of a two-input temporal AND gate with four distinct models. (a) Cooperative hybridization modeled as trimolecular reactions. After the second signal (A or B) has arrived, a second memory strand (a or b) will be produced at a relatively small amount due to the competition between memory formation ($A + GA \to a$ or $B + GB \to b$) and output production ($a + B + GaB \to Y$ or $b + A + GbA \to Z$). The release of the memory strand consumes a fraction of the signal and thus lowers the amount of the desired output, but it does not lead to any undesired output. (b) Cooperative hybridization modeled as trimolecular reactions; crosstalk included. With crosstalk, the second memory strand will react with the first to cooperatively produce undesired output ($a+b+GaB \rightleftharpoons Y + GB'$ or $b + a + GbA \rightleftharpoons Z + GA'$). (c) Cooperative hybridization modeled as bimolecular reactions; crosstalk included. When modeled as bimolecular reactions, only a fraction of the first memory strand will remain free, and the rest will be bound to a cooperative gate either in the desired pathway (a:GaB or b:GbA) or in the crosstalk pathway (GbA:a or GaB:b). The reduced amount of both reactants in cooperative hybridization and the likely chance that they will be bound to two different copies of a cooperative gate result in slower output production. (d) Cooperative hybridization modeled as bimolecular reactions; crosstalk and toehold occlusion included. Toehold occlusion further decreases the amount of free signal and memory strands, which results in even slower kinetics and more crosstalk.

S3 Supplementary simulation and data

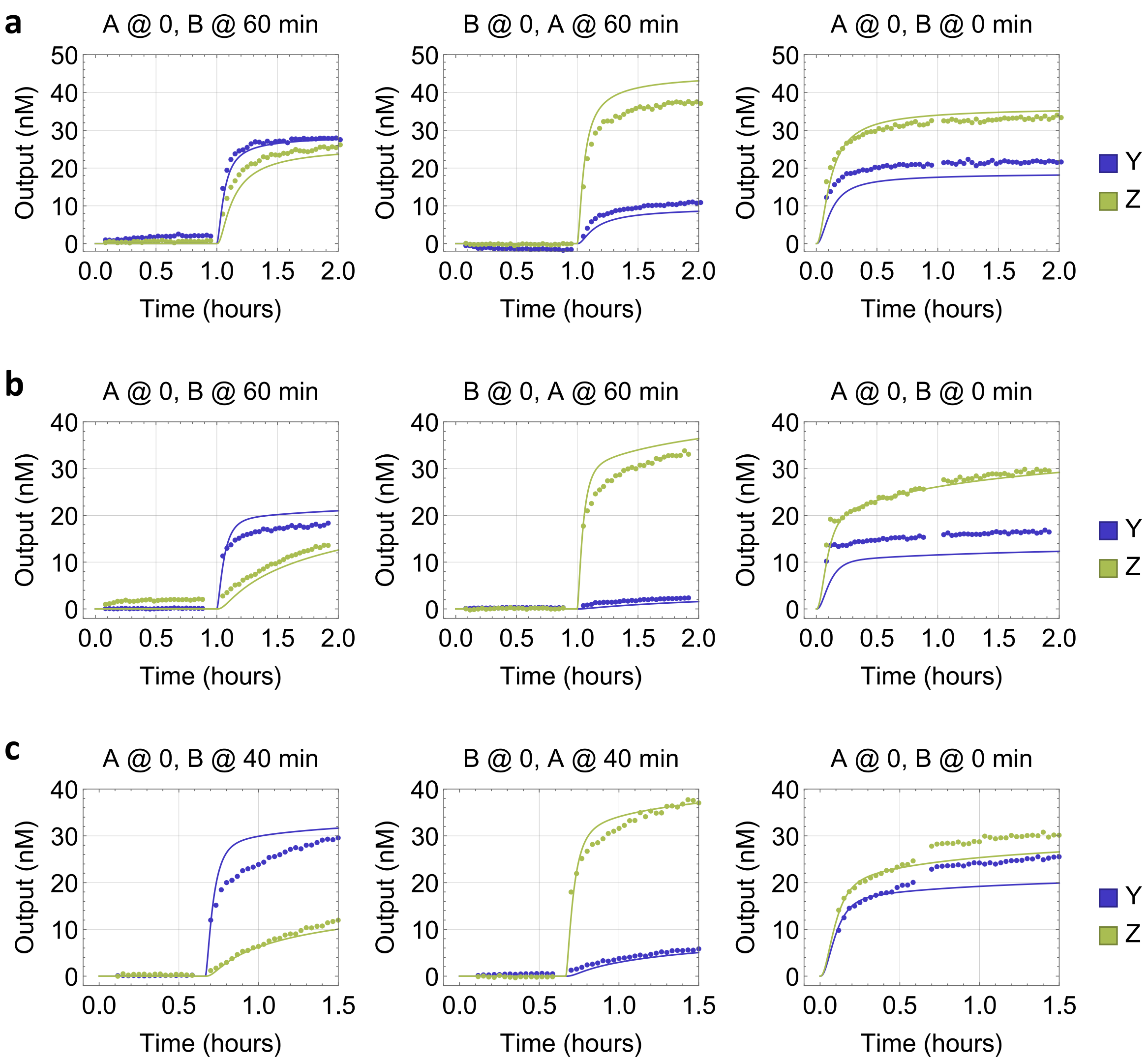

Figure S3: Simulation and fluorescence kinetics data of the two-input temporal AND gate without and with a mismatch in each of the memory strands. (a) No mismatch. The sequence of domain B is ACTCCTAATAT. (b) Mismatch. The sequence of domain B is ACTCCTAATAT and B′ is ACTCCTAATAA. (c) Mismatch. The sequence of domain B is TCAATCAACAC and B′ is TCAATCAACAA. All other domain sequences are the same as shown in Figure 4a. The plots in c are the same as in Figure 4b. All gates, reporters, and inputs were at 100, 150, and 90 nM, respectively.

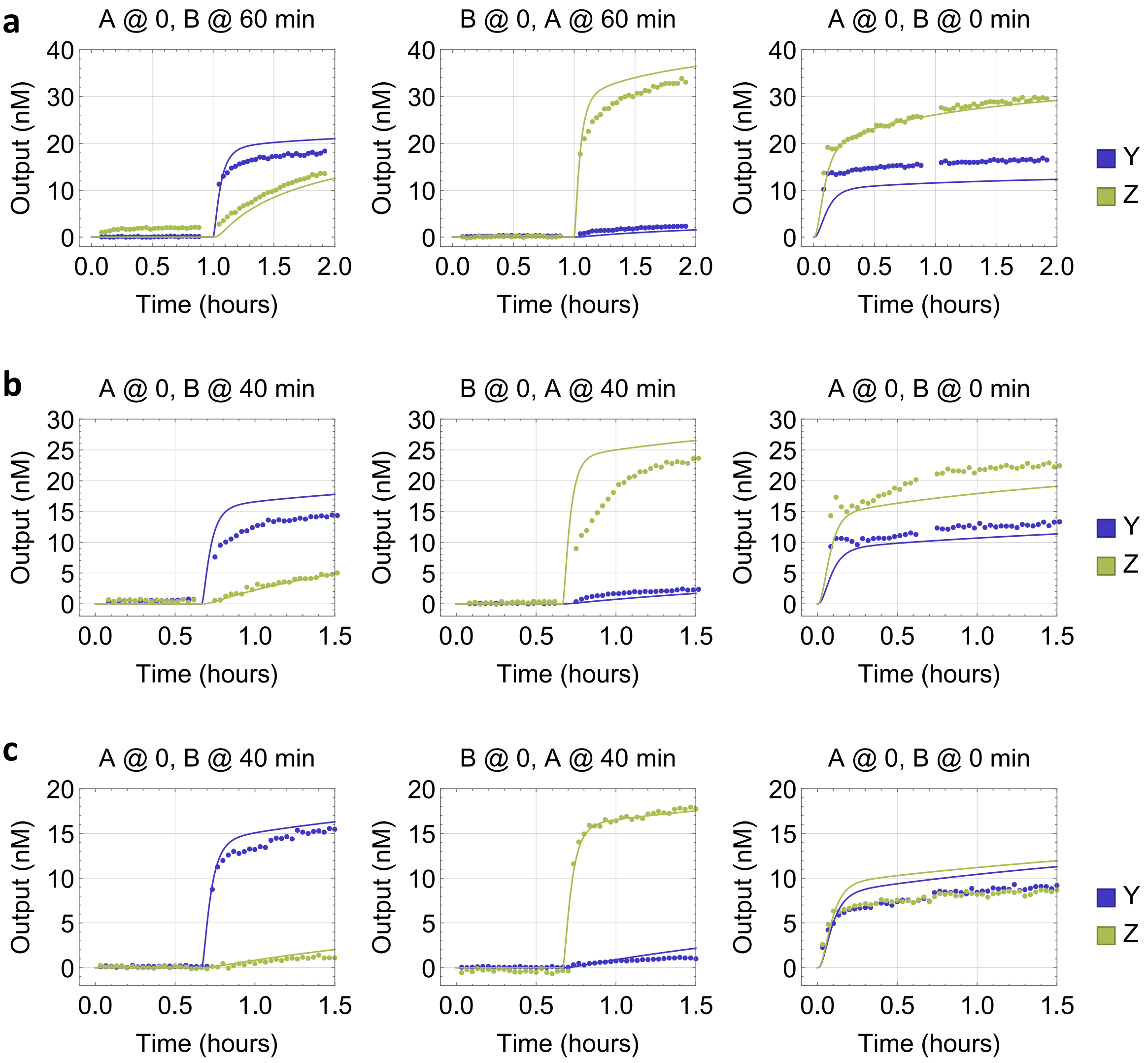

Figure S4: Simulation and fluorescence kinetics data of the two-input temporal AND gate with varying toehold lengths. (a) 7-nt, (b) 6-nt, and (c) 5-nt toehold S∗ on the 5' end of the bottom strand in cooperative gates aB and bA. The plots in a are the same as in Figure S3b. The plots in c are the same as in Figure 5c. All gates, reporters, and inputs were at 100, 150, and 90 nM, respectively.

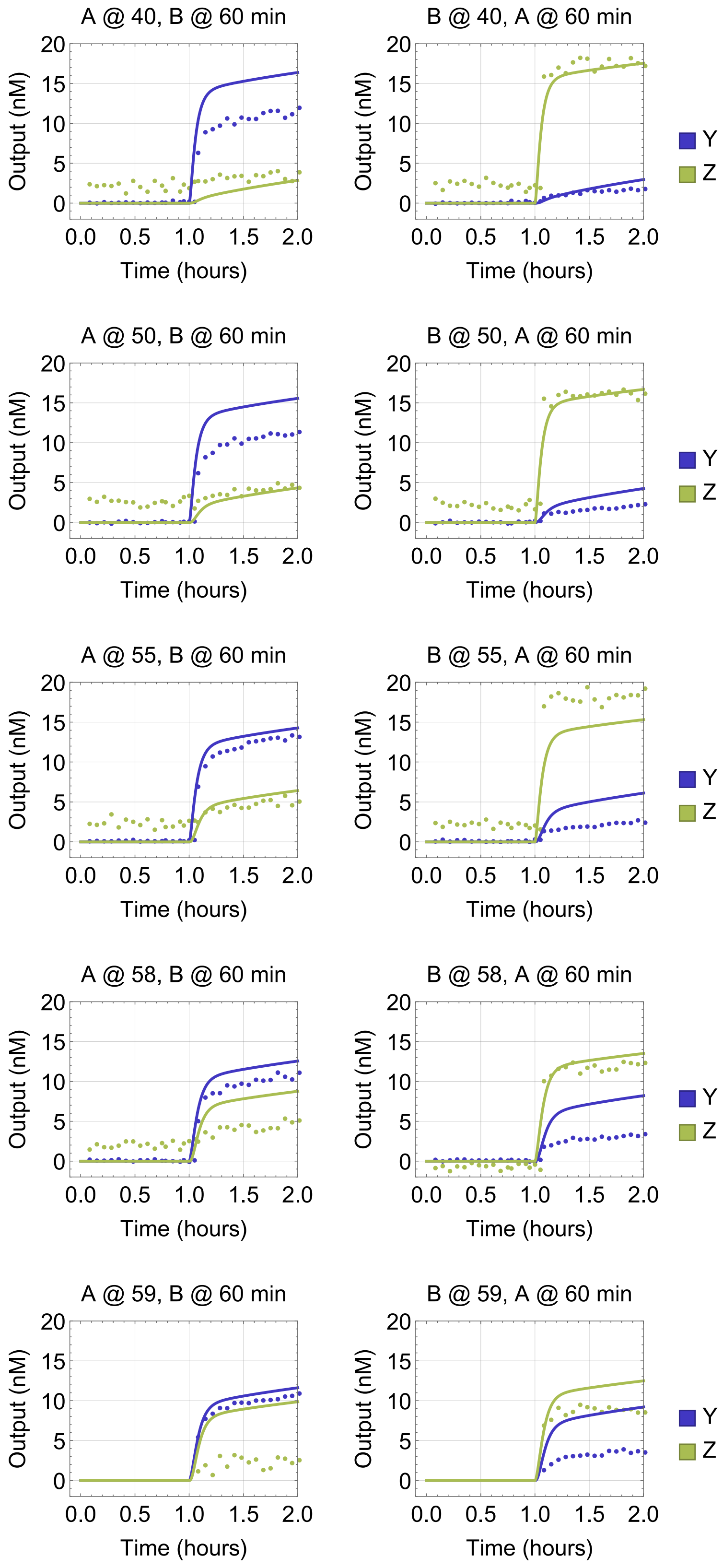

Figure S5: Simulation and fluorescence kinetics data of the two-input temporal AND gate with varying time intervals between inputs. All gates, reporters, and inputs were at 100, 150, and 90 nM, respectively. For data shown on the last row ($\Delta t = 1$ min), no measurements were collected before both inputs were added.

$$X_1 \xrightarrow{k_s} x_1$$
$$X_2 \xrightarrow{k_s} x_2$$
$$X_3 \xrightarrow{k_s} x_3$$

$$x_1 + X_2 \xrightarrow{k_m} x_{12}$$
$$x_1 + X_3 \xrightarrow{k_m} x_{13}$$
$$x_2 + X_1 \xrightarrow{k_m} x_{21}$$
$$x_2 + X_3 \xrightarrow{k_m} x_{23}$$
$$x_3 + X_1 \xrightarrow{k_m} x_{31}$$
$$x_3 + X_2 \xrightarrow{k_m} x_{32}$$

$$x_{12} + X_3 \xrightarrow{k_f} Y_{123}$$
$$x_{13} + X_2 \xrightarrow{k_f} Y_{132}$$
$$x_{21} + X_3 \xrightarrow{k_f} Y_{213}$$
$$x_{23} + X_1 \xrightarrow{k_f} Y_{231}$$
$$x_{31} + X_2 \xrightarrow{k_f} Y_{312}$$
$$x_{32} + X_1 \xrightarrow{k_f} Y_{321}$$

$$k_f \times c \gg k_m \times c \gg k_s$$

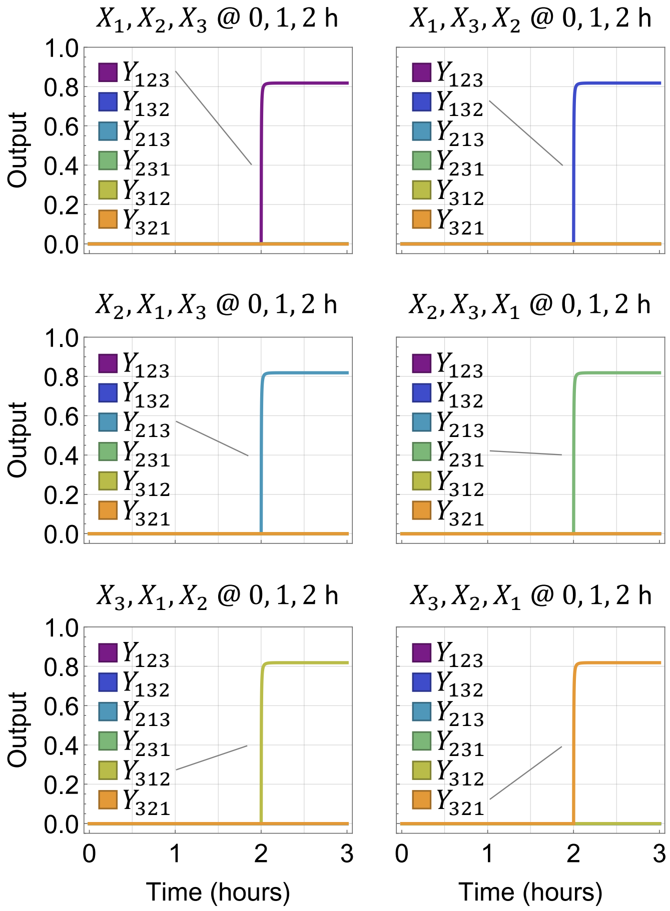

Figure S6: Chemical reaction network implementation of a three-input temporal AND gate. c is the concentration of input signals X_1 to X_3. Simulations of output signals Y_{123} to Y_{321} are shown as relative concentrations to c over time, where $c = 100$ nM, $k_s = 0.002$ /s, $k_m = 2 \times 10^5$ /M/s, and $k_f = 2 \times 10^6$ /M/s.

S4 DNA sequences

Table S1: DNA sequences of a two-input temporal AND gate without mismatches.

Name	Sequence
Signal A	CA TCT CA TATCTAATCTC CA CACTA CA
Signal B	CA TCT CA ACTCCTAATAT CA CACTA CA
Gate A-t (Signal a)	CA TATCTAATCTC CA CACTA CA TAACACAATCA
Gate A-b	TG TAGTG TG GAGATTAGATA TG AGA TG
Gate B-t (Signal b)	CA ACTCCTAATAT CA CACTA CA AATCTTCATCC
Gate B-b	TG TAGTG TG ATATTAGGAGT TG AGA TG
Gate aB-t (Signal Y)	CA TAACACAATCA CA TCT CA ACTCCTAATAT CA
Gate aB-b	TG TAGTG TG ATATTAGGAGT TG AGA TG TGATTGTGTTA TG TAGTG TG
Gate bA-t (Signal Z)	CA AATCTTCATCC CA TCT CA TATCTAATCTC CA
Gate bA-b	TG TAGTG TG GAGATTAGATA TG AGA TG GGATGAAGATT TG TAGTG TG

Table S2: DNA sequences of a two-input temporal AND gate with mismatches.

Name	Sequence
Signal A	CA TCT CA TATCTAATCTC CA CACTA CA
Signal B	CA TCT CA ACTCCTAATAT CA CACTA CA
Gate A-t (Signal a)	CA TATCTAATCTA CA CACTA CA TAACACAATCA
Gate A-b	TG TAGTG TG GAGATTAGATA TG AGA TG
Gate B-t (Signal b)	CA ACTCCTAATAA CA CACTA CA AATCTTCATCC
Gate B-b	TG TAGTG TG ATATTAGGAGT TG AGA TG
Gate aB-t (Signal Y)	CA TAACACAATCA CA TCT CA ACTCCTAATAT CA
Gate aB-b	TG TAGTG TG ATATTAGGAGT TG AGA TG TGATTGTGTTA TG TAGTG TGT
Gate bA-t (Signal Z)	CA AATCTTCATCC CA TCT CA TATCTAATCTC CA
Gate bA-b	TG TAGTG TG GAGATTAGATA TG AGA TG GGATGAAGATT TG TAGTG TGT

Table S3: DNA sequences of a two-input temporal AND gate with mismatches and an alternative domain B.

Name	Sequence
Signal A	CA TCT CA TATCTAATCTC CA CACTA CA
Signal B	CA TCT CA TCAATCAACAC CA CACTA CA
Gate A-t (Signal a)	CA TATCTAATCTC CA CACTA CA TAACACAATCA
Gate A-b	TG TAGTG TG GAGATTAGATA TG AGA TG
Gate B-t (Signal b)	CA TCAATCAACAA CA CACTA CA AATCTTCATCC
Gate B-b	TG TAGTG TG GTGTTGATTGA TG AGA TG
Gate aB-t (Signal Y)	CA TAACACAATCA CA TCT CA TCAATCAACAC CA
Gate aB-b	TG TAGTG TG GTGTTGATTGA TG AGA TG TGATTGTGTTA TG TAGTG TGT
Gate bA-t (Signal Z)	CA AATCTTCATCC CA TCT CA TATCTAATCTC CA
Gate bA-b	TG TAGTG TG GAGATTAGATA TG AGA TG GGATGAAGATT TG TAGTG TGT

Table S4: DNA sequences of a two-input temporal AND gate with mismatches and varying toehold lengths.

Name	Sequence
Signal A	CA TCT CA TATCTAATCTC CT ATACC CA
Signal B	CA TCT CA ACTCCTAATAT CA CACTA CA
Gate A-t (Signal a)	CA TATCTAATCTA CT ATACC CA TAACACAATCA
Gate A-b	TG GGTAT AG GAGATTAGATA TG AGA TG
Gate B-t (Signal b)	CA ACTCCTAATAT CA CACTA CA AATCTTCATCC
Gate B-b	TG TAGTG TG ATATTAGGAGT TG AGA TG
Gate aB-t (Signal Y)	CA TAACACAATCA CA TCT CA ACTCCTAATAT CA
Gate aB-b 7ntToe	TG TAGTG TG ATATTAGGAGT TG AGA TG TGATTGTGTTA TG GGTAT AGT
Gate aB-b 6ntToe	G TAGTG TG ATATTAGGAGT TG AGA TG TGATTGTGTTA TG GGTAT AGT
Gate aB-b 5ntToe	TAGTG TG ATATTAGGAGT TG AGA TG TGATTGTGTTA TG GGTAT AGT
Gate bA-t (Signal Z)	CA AATCTTCATCC CA TCT CA TATCTAATCTC CT
Gate bA-b 7ntToe	TG GGTAT AG GAGATTAGATA TG AGA TG GGATGAAGATT TG TAGTG TGT
Gate bA-b 6ntToe	G GGTAT AG GAGATTAGATA TG AGA TG GGATGAAGATT TG TAGTG TGT
Gate bA-b 5ntToe	GGTAT AG GAGATTAGATA TG AGA TG GGATGAAGATT TG TAGTG TGT

Table S5: DNA sequences of reporters.

Name	Sequence
Reporter Y-t	/5IAbRQ/ CATAACACAATCACA
Reporter Y-b	TG AGA TGTGATTGTGTTATG /3ATTO590N/
Reporter Z-t	/5IABkFQ/ CAAATCTTCATCCCA
Reporter Z-b	TG AGA TGGGATGAAGATTTG /3ATTO488N/

References

[1] Qian, L. & Winfree, E. Scaling up digital circuit computation with DNA strand displacement cascades. *Science* **332**, 1196–1201 (2011).

[2] Thubagere, A. J. *et al.* Compiler-aided systematic construction of large-scale DNA strand displacement circuits using unpurified components. *Nature Communications* **8**, 1–12 (2017).

[3] Qian, L. Seesaw Compiler. `http://qianlab.caltech.edu/SeesawCompiler` (2011).

[4] Zadeh, J. N. *et al.* NUPACK: analysis and design of nucleic acid systems. *Journal of Computational Chemistry* **32**, 170–173 (2011).

[5] Srinivas, N., Parkin, J., Seelig, G., Winfree, E. & Soloveichik, D. Enzyme-free nucleic acid dynamical systems. *Science* **358** (2017).

[6] Cherry, K. M. & Qian, L. Scaling up molecular pattern recognition with DNA-based winner-take-all neural networks. *Nature* **559**, 370–376 (2018).

[7] Soloveichik, D. CRNSimulator. `http://users.ece.utexas.edu/~soloveichik/crnsimulator.html` (2009).
